# Plasma Soluble Urokinase Receptor Level Is Correlated with Podocytes Damage in Patients with IgA Nephropathy

**DOI:** 10.1371/journal.pone.0132869

**Published:** 2015-07-13

**Authors:** Yanfeng Zhao, Lijun Liu, Jing Huang, Sufang Shi, Jicheng Lv, Gang Liu, Minghui Zhao, Hong Zhang

**Affiliations:** 1 Renal Division, Department of Medicine, Peking University First Hospital, Beijing, China; 2 Peking University Institute of Nephrology, Beijing, China; 3 Key Laboratory of Renal Disease, Ministry of Health of China, Beijing, China; 4 Key Laboratory of Chronic Kidney Disease Prevention and Treatment (Peking University), Ministry of Education, Beijing, China; University of Utah School of Medicine, UNITED STATES

## Abstract

**Background:**

Focal segmental glomerulosclerosis (FSGS) lesions are similar in characteristics to S lesions of the Oxford classification of IgA nephropathy (IgAN) and may predict poor prognosis. In the present study, we aimed to explore the association between plasma soluble urokinase receptor (suPAR) levels and S lesions and podocytes damage in IgAN patients.

**Methods:**

We enrolled 569 IgAN patients with follow-up data and detected plasma suPAR levels at renal biopsy by enzyme-linked immunosorbent assay.

**Results:**

Plasma suPAR levels in IgAN patients with or without S lesions did not differ significantly (*P* = 0.411). However, suPAR levels were positively correlated with proteinuria (r = 0.202, *P* < 0.001), and negatively correlated with estimated glomerular filtration rate (eGFR, r = –0.236, *P* < 0.001). In the partial correlation to adjust for eGFR, plasma suPAR levels remained positively correlated with proteinuria (r = 0.112, *P* = 0.023). In a Cox proportional hazards model, higher levels of plasma suPAR were not associated with poor renal outcome. Plasma suPAR levels of IgAN and primary FSGS patients with nephrotic syndrome were not significantly different (*P* = 0.306). Plasma suPAR levels in patients with extensive effacement of the epithelial cell foot processes of glomerular podocytes were significantly higher than those with segmental effacement on the basis of comparable eGFR (*P* = 0.036).

**Conclusions:**

In IgAN patients, plasma suPAR levels were not associated with S lesions. However, they were positively associated with proteinuria and negatively associated with eGFR. In addition, plasma suPAR levels were positively associated with the effacement degree of the foot processes, which might partially contribute to the development of proteinuria in patients with IgAN.

## Introduction

Immunoglobulin A nephropathy (IgAN), characterized by IgA deposition in glomerular mesangium, is the most common form of primary glomerulonephritis worldwide [1]. Patients with IgAN may present with a variety of different histological patterns, ranging from minimal glomerular lesions to diffuse crescentic glomerulonephritis by routine light microscopy [2–4]. The Oxford classification, composed of four histopathologic features (mesangial hypercellularity [M], endocapillary hypercellularity [E], segmental glomerulosclerosis/adhesion [S], and tubular atrophy and interstitial fibrosis [T]) is the most popular scoring system for predicting renal prognosis of IgAN independent of clinical features [5, 6]. More specifically, many previous studies have confirmed that S lesions can independently predict the loss of estimated glomerular filtration rate (eGFR) and lower renal survival [7–10].

A series of studies have described that lesions with a similar morphological form to focal segmental glomerulosclerosis (FSGS) may appear in IgAN [11–13]. Haas et al. included FSGS as one of the classes in his classification of IgAN even though this term has been superseded in the Oxford classification by the term S lesion [5–7, 14]. IgAN patients with FSGS had significantly faster decline in eGFR and worse renal survival than those without FSGS [12, 13, 15]. The FSGS-like lesion is not completely equivalent to the S lesion of the Oxford classification, because the latter includes all segmental scars and capsular adhesions, especially scars of unknown origin [15]. Nevertheless, segmental glomerulosclerosis is the common pathological manifestation of these lesions. Hence, there is likely a similar pathogenesis underlying both diseases.

Despite this controversy, soluble urokinase receptor (suPAR) is regarded as a circulating pathogenic factor in patients with primary FSGS [16]. Elevated suPAR levels can induce activation of podocyte β3 integrin, thereby causing foot process effacement and proteinuria, which is regarded as a key event in the initiation of proteinuric glomerular disease. Huang J et al. also found that in patients with primary FSGS achieving remission during the follow-up, plasma suPAR levels decreased significantly [17].

Thus, suPAR in circulation may participate in the pathogenesis, affect treatment response, and predict the prognosis of primary FSGS. As previously mentioned, the S lesion of the Oxford classification is similar to FSGS. Therefore, we investigated whether IgAN patients with S lesions could be differentiated from IgAN patients by measuring plasma suPAR levels, and to explore the association between plasma suPAR levels and IgAN pathogenesis, treatment response, and prognosis.

## Materials and Methods

### Study population

In total, 569 IgAN patients with regular follow-up of at least 12 months at Peking University First Hospital were enrolled in the present study. IgAN diagnosis was based on the presence of dominant IgA demonstration in the mesangial area by immunofluorescence, and confirmed by optical and electronic microscopy, and the lack of clinical or serological evidence of other inflammatory diseases, such as systemic lupus erythematosus, vasculitis, or Henoch–Schoenlein purpura. During follow-up, patients received the same treatment strategy according to the Kidney Disease: Improving Global Outcomes (KDIGO) Guideline. If proteinuria was >1 g/day, ACEI or ARBs were provided and dosages were adjusted according to changes of blood pressure with the optimal target of <130/80 mmHg. If proteinuria was >1 g/day for 3 to 6 months without relief, and eGFR was higher than 50 mL/min/1.73m^2^, steroids are recommended. Steroids, in combination with other immunosuppressive agents, like cyclophosphamide, mycophenolate mofetil, or FK506, were prescribed for crescentic IgA nephropathy with rapid progression [18, 19].

For enrolled patients, clinical manifestations, including age, gender, blood pressure, C reactive protein, plasma albumin, serum creatinine, and 24 hour urine protein excretion were collected from medical records at the time of renal biopsy. eGFR was calculated using the Chronic Kidney Disease Epidemiology Collaboration equation [20]. All renal biopsy specimens were reviewed and graded by two independent pathologists who were blinded to patient data and outcomes.

Moreover, 14 patients with MCD, 29 patients with membranous nephropathy MN, 74 patients with primary FSGS, and 14 patients with secondary FSGS were used as disease controls. The pathologic diagnosis and treatment strategy for primary FSGS and secondary FSGS have been reported in our previous study [17]. We also detected plasma suPAR levels of 86 age- and gender-matched normal subjects as healthy controls.

The study protocol was approved by the Medical Ethics Committee of Peking University First Hospital and informed written consent was obtained from every participant.

### Definition of clinical terms

The Oxford classification of the individual patients was analyzed, which were defined by four pathological features: mesangial hypercellularity score (M; M0 ≤0.5, M1 >0.5), the presence of endocapillary proliferation (E; E0: absent, E1: present), segmental glomerulosclerosis/adhesion (S; S0: absent, S1: present), and severity of tubular atrophy/interstitial fibrosis (T; T0: 25%, T1: 26%–50%, T2>50%) [5].

The composite end point, defined as a 50% eGFR decline, ESRD or death, whichever occurred first, was used in the present study. ESRD was defined as eGFR < 15 mL/min per 1.73 m^2^ or need for renal replacement therapy (such as hemodialysis, peritoneal dialysis, or renal transplantation), for the purpose of this study.

We defined effacement degrees of <50%, 50%–90%, >90% of the epithelial cell foot processes detected by electronic microscopy as segmental, most, and extensive effacement, respectively.

### Detection of plasma suPAR by ELISA

Early morning plasma samples from patients were collected on the day of renal biopsy using disodium-ethylene diamine tetraacetic acid (EDTA) as an anticoagulant. The plasma samples from 86 age- and gender-matched healthy donors were collected as normal controls. All samples were centrifuged immediately at 2000 *g* for 15 minutes at 4°C. Supernatants were stored at –80°C until assaying. Repeated freeze/thaw cycles were avoided. Plasma suPAR was quantified by the Quantikine Human uPAR Immunoassay (R&D Systems), according to the manufacturer’s protocol. The detailed five-step procedure has been shown in our preview study [17].

### Statistical analyses

Statistical analyses were performed by SPSS software (version 16.0; SPSS, Chicago, IL, USA). Normally distributed quantitative variables are expressed as means ± standard deviation. For non-normally distributed variables, we used median and IQR. Categorical data are summarized as absolute frequencies and percentages. For Continuous variables, independent-samples *t*-test was used if the data was normally distributed, and if not, Mann-Whitney or Kruskal-Wallis tests were performed. Categorical variables were compared using the χ^2^ test. Spearman’s correlation was applied for analyzing correlations. We used Cox proportional hazard models to analyze the association of plasma suPAR levels and composite outcome. Results are presented as HR and 95%CI. A two-tailed *P*-value less than 0.05 was considered statistically significant.

## Results

### Baseline characteristics

The baseline characteristics of patients with IgAN (n = 569) are shown in Table 1. The mean age at renal biopsy for these patients was 34.51 ± 11.97 years. Among these patients, 286 (50.3%) were males and 283 (49.7%) were females. Median proteinuria was 1.57 g/day and average eGFR was 83.65 ± 28.61 mL/min/1.73m^2^. Mean systolic blood pressure (SBP) and diastolic blood pressure (DBP) were 123 ± 15 mmHg and 79 ± 11 mmHg, respectively. Mesangial hypercellularity (M1), endocapillary hypercellularity (E1), and segmental glomerulosclerosis/adhesion (S1) were found in 75.7%, 59.6%, and 71.0% of patients, respectively. Tubular atrophy and interstitial fibrosis, 0% to 25% (T0), 26% to 50% (T1), and >50% (T2), were found in 65.9%, 21.4%, and 12.6% of patients, respectively. Average plasma suPAR level was 2467.08 pg/mL. All patients were regularly followed up, with a median follow-up time of 52 months. During the follow-up period, 551 (96.8%) patients received angiotensin-converting enzyme inhibitors (ACEI) or angiotensin receptor blocker (ARB) therapy, 261 (45.9%) received oral corticosteroids alone or combined with other immunosuppressive agents. In total, 86 patients reached the composite end point of a 50% decline in eGFR (n = 64), end-stage renal disease (ESRD, n = 61; 39 had a 50% eGFR decline with ESRD), or death (n = 4; one had a 50% decline in eGFR before death).

**Table 1:** Demographics and plasma suPAR levels in patients with IgAN.

	Mean ± SD or Median (IQR)
**Baseline**	
Age (years)	34.51 ± 11.97
Gender (male / female)	286 (50.3%) / 283 (49.7%)
suPAR value (pg/ml)	2467.08 ± 983.71
Initial proteinuria (g/day)	1.57 (0.87, 3.03)
<0.3 (%)	25/567 (4.4%)
0.3–0.99 (%)	145/567 (25.6%)
1.0–2.99 (%)	254/567 (44.8%)
≥3.0 (%)	143/567 (25.2%)
eGFR (ml/ min per 1.73 m^2^)	83.65 ± 28.61
CKD Stages 1, 2, 3, and 4 ^a^	258(45.3%), 192(33.7%), 99(17.4%), 20(3.5%)
SBP (mmHg)	123 ± 15
DBP (mmHg)	79 ± 11
Oxford classification ^b^	
M1	420 (75.7%)
E1	331 (59.6%)
S1	394 (71.0%)
T1/T2	119 (21.4%) / 70 (12.6%)
**During follow-up period**	
Follow-up interval (months)	51.85 ± 29.24
Therapy of ACE inhibitors or ARBs	551 (96.8%)
Therapy of prednisone and any other immunosuppressive agents (cyclophosphamide, MMF, or others)	261 (45.9%)

Abbreviations: IgAN, IgA nephropathy; SD, standard deviation; IQR, interquartile range; CKD, chronic kidney disease; SBP, systolic blood pressure; DBP, diastolic blood pressure; eGFR, estimate glomerular filtration rate; ACE, angiotensin-converting enzyme; ARB, angiotensin II receptor blocker; MMF, mycophenolate mofetil. Oxford classification: mesangial hypercellularity score (M1 >0.5), the presence of endocapillary proliferation (E1: present), segmental glomerulosclerosis/adhesion (S1: present), and severity of tubular atrophy/interstitial fibrosis (T1: 26%–50%, T2 >50%).

^a^ CKD stage 1, 2, 3, and 4 were divided by eGFR ≥ 90, 60–89, 30–59, and 15–29 ml/ min per 1.73 m^2^, respectively, according to KDOQI.

^b^ Oxford classification was developed by Working Group of the International IgA Nephropathy Network and the Renal Pathology Society.

### Plasma suPAR levels correlated with IgAN severity

A cross section correlation analysis between plasma suPAR levels and clinical and histological manifestations of IgAN patients at renal biopsy was performed (Table 2). The patients with higher initial proteinuria showed higher plasma suPAR levels (<0.3 g/d: 2232.54 ± 1059.42 pg/mL, 0.3–0.99 g/d: 2197.82 ± 763.13 pg/mL, 1.0–2.99 g/d: 2502.21 ± 971.18 pg/mL, and ≥3.0 g: 2718.08 ± 1117.88 pg/mL, respectively, *P* < 0.001). Patients with worse renal function showed higher plasma suPAR levels (CKD1: 2295.44 ± 934.52 pg/mL, CKD2: 2463.39 ± 891.17 pg/mL, CKD3: 2840.52 ± 1147.60 pg/mL, and CKD4: 2868.23 ± 1021.14 pg/mL, respectively, *P* < 0.001). Plasma suPAR levels in patients with M lesions were higher than those without M lesions (2534.70 ± 1017.86 pg/mL vs. 2245.97 ± 800.03 pg/mL, *P* = 0.001). Patients with more serious T lesions showed higher plasma suPAR levels (T0: 2353.41 ± 935.16 pg/mL, T1: 2519.58 ± 881.87 pg/mL, and T2: 2951.49 ± 1179.15 pg/mL; *P* < 0.001). Conversely, plasma suPAR levels in patients with or without S lesions were not significantly different (2411.13 ± 971.62 pg/mL vs. 2486.27 ± 979.03 pg/mL, *P* = 0.411).

**Table 2:** Comparison of plasma suPAR levels between subgroups of variable clinical or histologic parameters in patients with IgAN.

Parameters	suPAR (pg/mL)	P Value
Gender		0.073
Male	2393.65±957.10	
Female	2541.29±1006.13	
Initial proteinuria (g/day)		<0.001
<0.3	2232.54±1059.42	
0.3–0.99	2197.82±763.13	
1.0–2.99	2502.21±971.18	
≥3.0	2718.08±1117.88	
CKD stage		<0.001
1	2295.44±934.52	
2	2463.39±891.17	
3	2840.52±1147.60	
4	2868.23±1021.14	
Oxford classification		
Mesangial hypercellularity		0.001
0	2245.97±800.03	
1	2534.70±1017.86	
Endocapillary hypercellularity		0.537
0	2495.66±970.80	
1	2443.37±981.42	
Segmental glomerulosclerosis		0.411
0	2411.13±971.62	
1	2486.27±979.03	
Tubular atrophy/interstitial fibrosis		<0.001
0	2353.41±935.16	
1	2519.58±881.87	
2	2951.49±1179.15	

Abbreviations: IgAN, IgA nephropathy; CKD, chronic kidney disease.

We also conducted a relative analysis of plasma suPAR and clinical manifestations. SuPAR levels in IgAN patients were positively correlated with proteinuria (r = 0.202, *P* < 0.001, Table 3), and negatively correlated with eGFR (r = –0.236, *P* < 0.001). Conversely, plasma suPAR levels weren’t related to C reactive protein (r = 0.027, *P* = 0.589). To exclude the influence of reduced renal function, we performed a partial correlation to adjust for eGFR. We found that plasma suPAR levels remained positively correlated with proteinuria (r = 0.112, *P* = 0.023) even after this correction.

**Table 3:** Correlation of plasma suPAR levels with clinical parameters in patients with IgAN.

	Before adjusting		After adjusting for eGFR	
Parameters	R Value	P Value	R Value	P Value
Age	0.130	0.002	0.025	0.615
Proteinuria	0.202	<0.001	0.112	0.023
eGFR	-0.236	<0.001	——	——
SBP	0.079	0.060	0.038	0.445
DBP	0.102	0.015	0.028	0.568
C reactive protein	0.027	0.589	0.019	0.702

Abbreviations: IgAN, IgA nephropathy; eGFR, estimate glomerular filtration rate; SBP, systolic blood pressure; DBP, diastolic blood pressure.

In the multivariable linear regression model, proteinuria, eGFR, and M and T were independently associated with plasma suPAR level (β = 0.121, *P* = 0.005; β = –0.120, *P* = 0.038; β = 0.103, *P* = 0.016, and β = 0.102, *P* = 0.048, respectively) after adjusting for variables, including age, gender, SBP, DBP, E lesion, and S lesion.

### Plasma suPAR levels don’t correlate with IgAN progression

In order to evaluate the prognostic effect of plasma suPAR for IgAN progression, we applied a multivariable linear regression model using kidney function decline slope as the dependent variable, and adjusted for age, gender, systolic blood pressure, diastolic blood pressure, initial proteinuria, and Oxford classification. The results indicate that plasma suPAR levels were not independently associated with kidney function decline (β = 0.019, *P* = 0.671, data not shown).

In a Cox proportional hazards model, we tested baseline clinical and pathological variables in order to explore an association with the composite end point. In univariate analyses, higher plasma suPAR levels were not significantly associated with a poor renal outcome (hazard ratio [HR], per standard deviation [s.d.] increment of natural log-transformed suPAR: 1.648; 95% confidence interval [CI]: 0.955–2.845, *P* = 0.073, Table 4). After adjusting for well-established risk factors for IgAN, higher levels of plasma suPAR were still not significantly associated with poor renal outcome (HR, per s.d. increment of natural log–transformed suPAR: 1.065; 95%CI: 0.609–1.861, *P* = 0.826).

**Table 4:** Risks of composite end-point of natural log–transformed suPAR.

		Hazard ratio (95% confidence interval) and *P* value		
	Unadjusted	Model 1^a^	Model 2^b^	Model 3^c^
Composite end-point	1.648 (0.955–2.845)	1.747 (1.006–3.035)	1.062 (0.604–1.866)	1.065 (0.609–1.861)
Per 1 s.d lnsuPAR	0.073	0.048	0.835	0.826

Composite end point was defined as a 50% decline of eGFR, end-stage renal disease, or death. Unadjusted model analyzed suPAR as continuous data.

^a^ Model 1 adjusted for gender and age. Gender was analyzed as dichotomous data.

^b^ Model 2 adjusted for covariates in model 1 plus estimate glomerular filtration rate (eGFR), initial proteinuria, systolic pressure and oxford classification (M, E, S, and T). The latter variable was analyzed as categorical data.

^c^ Model 3 adjusted for covariates in model 2 plus steroid or other immunosuppressants use (yes or no). The latter variable was analyzed as dichotomous data.

### Plasma suPAR levels in patients with IgAN and controls

**Fig 1:**
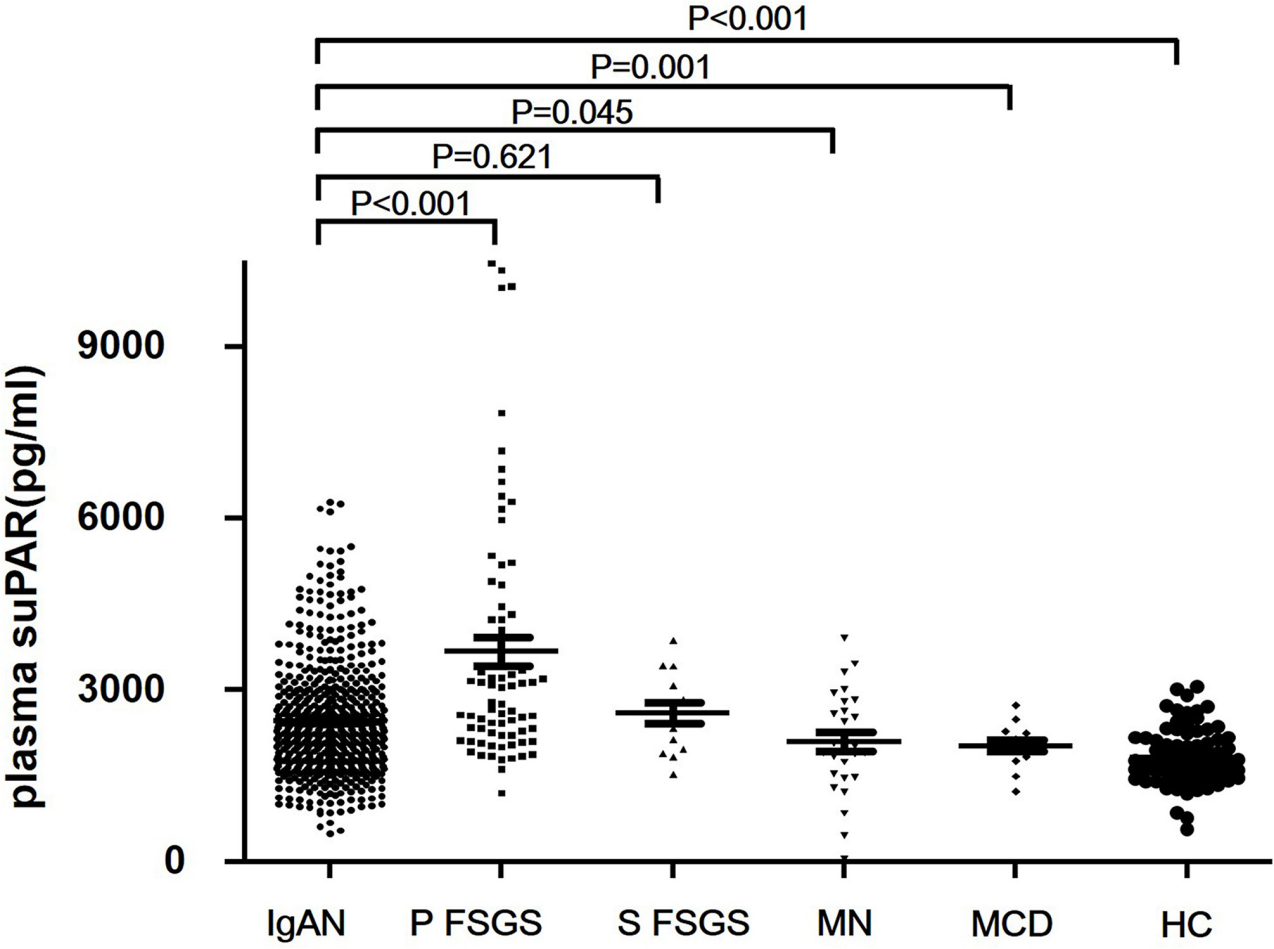
Plasma suPAR levels among patients with IgAN, primary FSGS, secondary FSGS, MN, MCD, and healthy controls. Plasma suPAR levels of patients with IgA nephopathy (IgAN, 2298, IQR 1776–2956 pg/mL) were significantly lower than patients with primary FSGS (FSGS, 2923, interquartile range (IQR) 2205–4360 pg/mL, *P* < 0.001), higher than those with membranous nephropathy (MN, 2028, IQR 1512–2715, *P* = 0.045), minimal change disease (MCD, 2050, IQR 1813–2249 pg/mL, *P* = 0.001), and healthy controls (HC, 1739, 1513–2121 pg/mL, *P* < 0.001), respectively. There was no significant difference in plasma suPAR levels between patients with IgA nephrology and patients with secondary FSGS (FSGS, 2639, IQR 1945–3166 pg/mL, *P* = 0.621).

Plasma suPAR levels in patients with IgAN, primary FSGS, secondary FSGS, minimal change disease (MCD), membranous nephropathy (MN), and healthy controls are shown in Table 5 and Fig 1 [17]. Plasma suPAR levels of patients with IgA nephropathy (2298, interquartile range [IQR] 1776–2956 pg/mL) were significantly lower than patients with primary FSGS (2923, IQR 2205–4360 pg/mL, *P* < 0.001), higher than those with MN (2028, IQR 1512–2715 pg/mL, *P* = 0.045), MCD (2050, IQR 1813–2249 pg/mL, *P* = 0.001) and healthy controls (1739, 1513–2121 pg/mL, *P* < 0.001). There were no significant differences in plasma suPAR levels between patients with IgAN and patients with secondary FSGS (2639, IQR 1945–3166 pg/mL, *P* = 0.621).

**Table 5:** Plasma suPAR levels in patients with IgAN and controls.

	IgA nephropathy	Primary FSGS	Secondary FSGS	Membranous nephropathy	Minimal change disease	Healthy control
Number of subjects	569	74	14	29	14	86
Age (median, range)	34, 32–42	29, 13–84	38, 14–46	50, 33–79	42, 17–71	36, 20–49
Gender (male/female)	286/283	50/24	5/9	18/11	7/7	48/38
Plasma suPAR (pg/mL) (median, IQR)	2298,	2923,	2639,	2028,	2050,	1739,
	1776–2956	2205–4360	1945–3166	1512–2715	1813–2249	1513–2121

Abbreviations: IgAN, IgA nephropathy; IQR, interquartile range.

Only eGFR of patients with IgA nephropathy were significantly higher than patients with primary FSGS (83.65 ± 28.61 mL/min/1.73 m^2^ vs. 63.64 ± 38.96 mL/min/1.73 m^2^, P < 0.001). And eGFR of patients with IgA nephropathy were comparable to secondary FSGS (73.69 ± 20.52 mL/min/1.73 m^2^, P = 0.097), MN (84.96 (76.00, 92.93) mL/min/1.73 m^2^, P = 0.600) and MCD (95.86 ± 34.10 mL/min/1.73 m^2^, P = 0.117). And, plasma suPAR levels were partially associated with the difference in kidney function to a certain extent.

**Fig 2:**
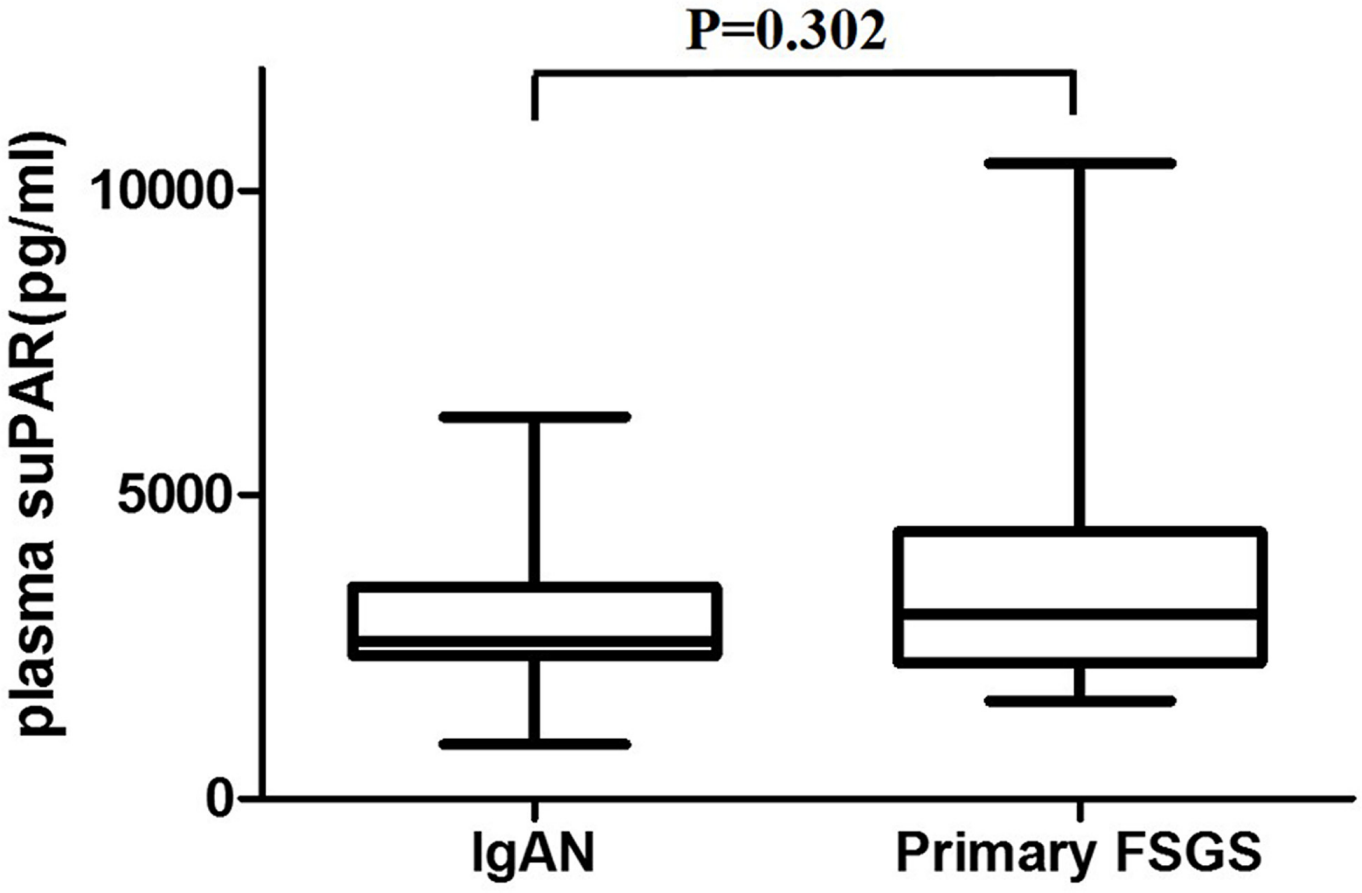
Plasma suPAR levels of IgAN and primary FSGS patients with nephrotic syndrome. The number of patients with nephrotic syndrome among IgAN and primary FSGS were 34 and 73, respectively. There was no significant difference in plasma suPAR levels between patients with IgAN (2590, IQR 2352–3489 pg/mL) and patients with primary FSGS (3049, 2233–4391 pg/mL, *P* = 0.306, Fig 2). eGFR didn’t show significant differences between groups (72.23 ± 39.96 mL/min/1.73 m^2^ vs. 83.32 ± 44.95 mL/min/1.73 m^2^, *P* = 0.223).

Overall, 73 of the 74 patients with primary FSGS had nephrotic syndrome, and there were 34 IgAN patients with nephrotic syndrome in our study. Among patients with nephrotic syndrome, we found no significant difference in plasma suPAR levels between IgAN (2590, IQR 2352–3489 pg/mL) and primary FSGS (3049, 2233–4391 pg/mL, *P* = 0.306, Fig 2). Moreover, eGFR wasn’t significantly different (72.23 ± 39.96 mL/min/1.73 m^2^ vs. 83.32 ± 44.95 mL/min/1.73 m^2^, *P* = 0.223).

### Plasma suPAR levels in patients with IgAN with different degree of epithelial cell damage

**Fig 3:**
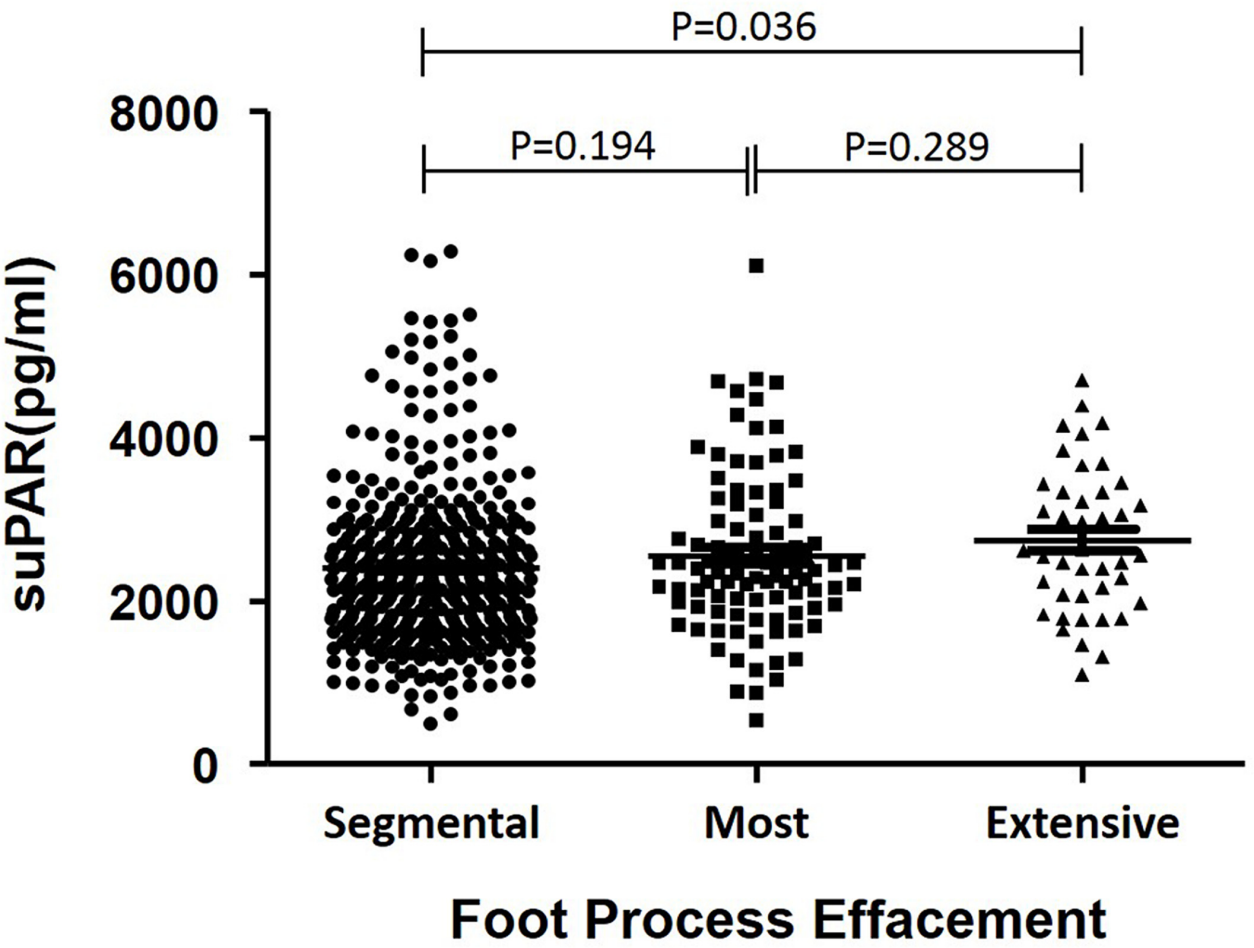
Plasma suPAR levels among IgAN patients with different degrees of effacement of foot processes. Epithelial cells were detected by electron microscopy and the numbers of patients with segmental, most, and extensive effacement of foot processes were 408, 96, and 42, respectively. We found that plasma suPAR levels in the extensive effacement group were significantly higher than those in the segmental effacement group (2741.57 ± 894.81 pg/mL vs. 2407.68 ± 987.88 pg/mL, *P* = 0.036, Fig 3). Moreover, eGFR was comparable between groups (81.29 ± 30.19 mL/min/1.73 m^2^ vs. 86.80 ± 26.60 mL/min/1.73 m^2^, *P* = 0.208).

In addition, we divided IgAN patients into three groups according to the degree of epithelial cell damage detected by electronic microscopy: segmental (n = 408), most (n = 96), and extensive (n = 42) effacement of foot processes, respectively. We found that plasma suPAR levels in the extensive effacement group were significantly higher than those in the segmental effacement group (2741.57 ± 894.81 pg/mL vs. 2407.68 ± 987.88 pg/mL, *P* = 0.036, Fig 3). Similarly, eGFR was not significantly different (81.29 ± 30.19 mL/min/1.73 m^2^ vs. 86.80 ± 26.60 mL/min/1.73 m^2^, *P* = 0.208).

## Discussion

IgAN is characterized by IgA deposition in the mesangial area and may manifest as a wide variation of clinical and pathologic presentations [2, 14, 21]. S lesions, one of the four histopathologic features of the Oxford classification, are well known for being an effective indicator of severity and bad prognosis in IgAN [7–10]. FSGS-like lesions, whose pathological characteristics are similar to S lesions, can accurately predict poor IgAN prognosis [15]. Despite controversy existing, some studies have revealed that serum suPAR levels are higher in primary FSGS than in secondary FSGS, MN, and MCD, and it may participate in the pathogenesis, affect treatment response, and predict the prognosis of patients with primary FSGS [16, 17]. In the present study, we aimed to explore whether the noninvasive marker, suPAR, could be used to evaluate IgAN patients with S lesions and prognosis.

uPAR is a glycosylphosphatidylinositol-anchored protein that can be cleaved to release soluble uPAR into plasma [22, 23]. It can be involved in non-proteolytic pathways based on its ability to interact with integrins and G protein-coupled receptors [23]. Our present work is the first clinical study to evaluate the relationship between plasma suPAR and S lesion and clinical data of IgAN in a cohort with a long-term follow-up. However, plasma suPAR levels were not higher in IgAN patients with S lesions. Possible reasons are as follows: Firstly, plasma suPAR is not involved in the pathogenesis of S lesions in IgAN [24–26]. Secondly, suPAR consists of three homologous domains, DI, DII, and DIII, which have various subunit configurations [27]. However, the commoditized enzyme-linked immunosorbent assay (ELISA) kits used in our study may not be able to distinguish full-length suPAR from other fragments, so non-pathogenic components might reduce the sensitivity of these tests. Thirdly, in addition to the real FSGS-like lesions, the S lesions in IgAN could also include post-adaptive S lesions, which are distinct from the suPAR induced primary podocytopathy in pathogenesis and usually manifest as segmental podocyte foot process effacement under electronic microscopy. In support of this, Fig 3 demonstrated that suPAR levels in patients with massive podocytopathy were significantly higher than those in patients with segmental podocytopathy.

SuPAR is a 20–50 kDa protein. SuPAR levels in plasma may be higher in patients with reduced eGFR. In this study, plasma suPAR levels were negatively associated with eGFR, which is in accordance with previous studies [24, 28–30]. After adjusting for eGFR, plasma suPAR levels remained positively correlated with M and T lesions. However, the molecular mechanisms underlying changes in mesangial cells and tubular cell functions by plasma suPAR are still poorly understood. In mesangial cells, Shushakova et al. showed that urokinase-type plasminogen activator (uPA) induces upregulated expression of the complement anaphylatoxin, and modulates C5a-dependent functional responses, resulting in upregulation of the C5a receptor via the specific receptor uPAR. Their study suggested a novel role for uPA/uPAR in masangial cell damage [31].

Podocytes, endothelial cells, and the glomerular basement membrane constitute the kidney filtration barrier, a highly specialized structure for selective ultrafiltration. Damage and detachment of podocytes can lead to foot processes retraction, thereby resulting in proteinuria. We divided IgAN patients into three groups according to the degree of podocyte damage, which were respectively described as segmental, most, and extensive effacement of foot processes by electronic microscopy. The group with extensive foot processes effacement had significantly higher plasma suPAR than the group with segmental effacement. Moreover, eGFR levels were comparable between the two groups, implying that plasma suPAR may participate in foot processes retraction, further engendering proteinuria. This result is consistent with the fact that plasma suPAR levels were independently related with 24 hour urinary protein excretion in multivariable linear regression analysis. We presume that plasma suPAR can induce pathological activation of podocyte β3 integrin, thereby causing foot processes effacement and resulting in proteinuria. Thus, plasma suPAR can cause podocyte damage and might not be a specific marker of primary FSGS, which is in agreement with previous studies [24–26, 28].

In a Cox regression model, we found that plasma suPAR couldn’t serve as an independent prognostic for composite IgAN end point. Therefore, we propose that plasma suPAR can lead to podocyte damage and consequently participate in IgAN pathogenesis, but does not accelerate IgAN progression directly.

Comparing plasma suPAR levels in IgAN patients with diseased and healthy controls, we found that they were significantly lower in patients with IgAN than in patients with primary FSGS. However, after matching eGFR, proteinuria, and plasma-albumin, there was no significant difference between the two groups. This demonstrates that IgAN patients with nephrotic syndrome might share the same underlying pathogenesis with primary FSGS, although this hypothesis requires further validation. In accordance with our assumption, the latest study of Joann M. Spinale revealed that after adjusting for eGFR and proteinuria, serum suPAR level was not an independent predictor of FSGS histopathology. Thus, Plasma suPAR may play the same role in various podocytopathies [32].

The specific mechanisms underlying circulating suPAR for inducing foot processes retraction remains unclear, and should be explored in future studies. Sequential plasma samples from our study patients were not available, and therefore we could not correlated plasma suPAR levels with therapy. The underlying mechanisms of the independent association between T lesions and plasma suPAR are also unknown. A previous study of Ji-Hye Lee, *et al*. revealed that uPA/uPAR expression on podocytes accompanies a decreased prevalence of T lesion, and in IgAN it suggests a possible protective effect of podocyte uPA/uPAR expression against interstitial fibrosis, which implied that uPAR expressed on podocyte protect tubulointerstitium through combination with uPA. However, plasma suPAR can induce pathological activation of receptor on podocytes, thereby causing kidney damage, including tubulointerstitial injury. The two mechanisms were different [33].

In conclusion, plasma suPAR levels did not correlate with S lesions of IgAN patients. Plasma suPAR levels were positively associated with proteinuria and negatively associated with eGFR, and associated with effacement degree of foot processes. A certain kind of IgAN patients might share the same pathogenesis of podocyte damage by suPAR as those with primary FSGS.
